# A nomogram for predicting survival in patients with gastrointestinal stromal tumor: a study based on the surveillance, epidemiology, and end results database

**DOI:** 10.3389/fmed.2024.1403189

**Published:** 2024-05-23

**Authors:** Xiaxi Li, Lijuan You, Qinghua Liu, Wenhua He, Xiaobing Cui, Wei Gong

**Affiliations:** Department of Gastroenterology, Shenzhen Hospital of Southern Medicine University, Shenzhen, China

**Keywords:** surveillance, epidemiology, and end results, cancer-specific survival, nomogram, gastrointestinal stromal tumor, predicting survival

## Abstract

**Purpose:**

The objective of this investigation was to construct and validate a nomogram for prognosticating cancer-specific survival (CSS) in patients afflicted with gastrointestinal stromal tumor (GIST) at 3-, 5-, and 8-years post-diagnosis.

**Methods:**

Data pertaining to patients diagnosed with GIST were acquired from the Surveillance, Epidemiology, and End Results (SEER) database. Through random selection, a training cohort (70%) and a validation cohort (30%) were established from the patient population. Employing a backward stepwise Cox regression model, independent prognostic factors were identified. Subsequently, these factors were incorporated into the nomogram to forecast CSS rates at 3-, 5-, and 8-years following diagnosis. The nomogram’s performance was assessed using indicators such as the consistency index (C-index), the area under the time-dependent receiver operating characteristic curve (AUC), the net reclassification improvement (NRI), the integrated discrimination improvement (IDI), calibration curves, and decision-curve analysis (DCA).

**Results:**

This investigation encompassed a cohort of 3,062 GIST patients. By analyzing the Cox regression model within the training cohort, nine prognostic factors were identified: age, sex, race, marital status, AJCC (American Joint Committee on Cancer) stage, surgical status, chemotherapy status, radiation status, and income status. The nomogram was subsequently developed and subjected to both internal and external validation. The nomogram exhibited favorable discrimination abilities, as evidenced by notably high C-indices and AUC values. Calibration curves confirmed the nomogram’s reliability. Moreover, the nomogram outperformed the AJCC model, as demonstrated by enhanced NRI and IDI values. The DCA curves validated the clinical utility of the nomogram.

**Conclusion:**

The present study has successfully constructed and validated the initial nomogram for predicting prognosis in GIST patients. The nomogram’s performance and practicality suggest its potential utility in clinical settings. Nevertheless, further external validation is warranted.

## 1 Introduction

Gastrointestinal stromal tumor (GIST) is a rare form of cancer that originates in the gastrointestinal tract (1). It primarily affects the connective tissue cells, known as stromal cells, found in the walls of the digestive system (2). GISTs can occur in various locations within the gastrointestinal tract, including the stomach, small intestine, and less commonly, the esophagus, colon, and rectum (3).

The incidence of GIST is relatively low compared to other gastrointestinal malignancies (4). However, it represents a distinct entity with unique characteristics that necessitate focused investigation and analysis (5). Despite its rarity, GIST has garnered significant attention due to its potential for aggressive behavior and variable clinical outcomes (6). To date, the understanding of GIST and its prognostic implications remains limited (7). To address this gap, researchers have explored potential risk factors and developed predictive models to aid in assessing patient prognosis and guiding treatment decisions (8).

One widely used tool in tumor prediction models is the nomogram, which provides a straightforward and accurate means of estimating a patient’s chances of survival based on various clinical and demographic factors (9). While nomograms have been established for several cancer types, including tonsil, parotid-gland, and breast cancer (10–12), the development of a nomogram specifically designed for GIST is yet to be reported. Consequently, there exists a need to construct a comprehensive nomogram for GIST patients, utilizing pertinent data from reliable sources such as the Surveillance, Epidemiology, and End Results (SEER) database.

The aim of our study was to develop and evaluate a novel nomogram for GIST patients, utilizing the extensive data available in the SEER database. This nomogram would incorporate key demographic information, clinicopathologic features, and therapeutic approaches to provide a personalized and thorough estimation of patient survival probabilities. By analyzing relevant treatment modalities, our nomogram would offer clinicians a valuable tool for guiding treatment decisions and optimizing patient outcomes.

The development of a specialized nomogram for GIST patients represents a significant advancement in personalized medicine. By incorporating essential patient characteristics and treatment approaches, this nomogram surpasses conventional methods, providing clinicians with a comprehensive and tailored approach to predicting patient survival. Through our study, we aimed to enhance the understanding of GIST and contribute to improved clinical decision-making for this distinct malignancy.

## 2 Patients and methods

### 2.1 Data sources and research factors

The SEER database was utilized, employing the SEER*Stat software, to filter and extract the relevant data. While a portion of the SEER database is accessible to the public, additional access to the SEER plus database was requested for comprehensive data retrieval (13). Gastrointestinal stromal tumor (GIST) cases were collected by applying the histology/behavior codes from the third revision of the International Classification of Diseases for Oncology (ICD-O-3), specifically “8935/3: Stromal sarcoma, NOS” and “8936/3: Gastrointestinal stromal sarcoma.” Furthermore, cases located in the digestive tract were selected for analysis.

Through a series of meticulous calculations and screenings using both univariate and multivariate Cox regression analyses, we identified a subset of these variables that demonstrated statistically significant associations with survival. The selection was guided by a rigorous statistical threshold to ensure that the included variables were not only statistically significant but also clinically meaningful. Age, race, sex, marital status, American Joint Committee on Cancer (AJCC) stage, income, summary of stage, surgery status, radiotherapy status, and chemotherapy status. Given the substantial multicollinearity arising from the inclusion of all these factors, the analysis focused solely on the AJCC staging system. The primary outcome variable of interest was cancer-specific survival (CSS). As the SEER database used in this study does not contain personally identifiable information, patient-informed consent was not required.

**Figure 1:**
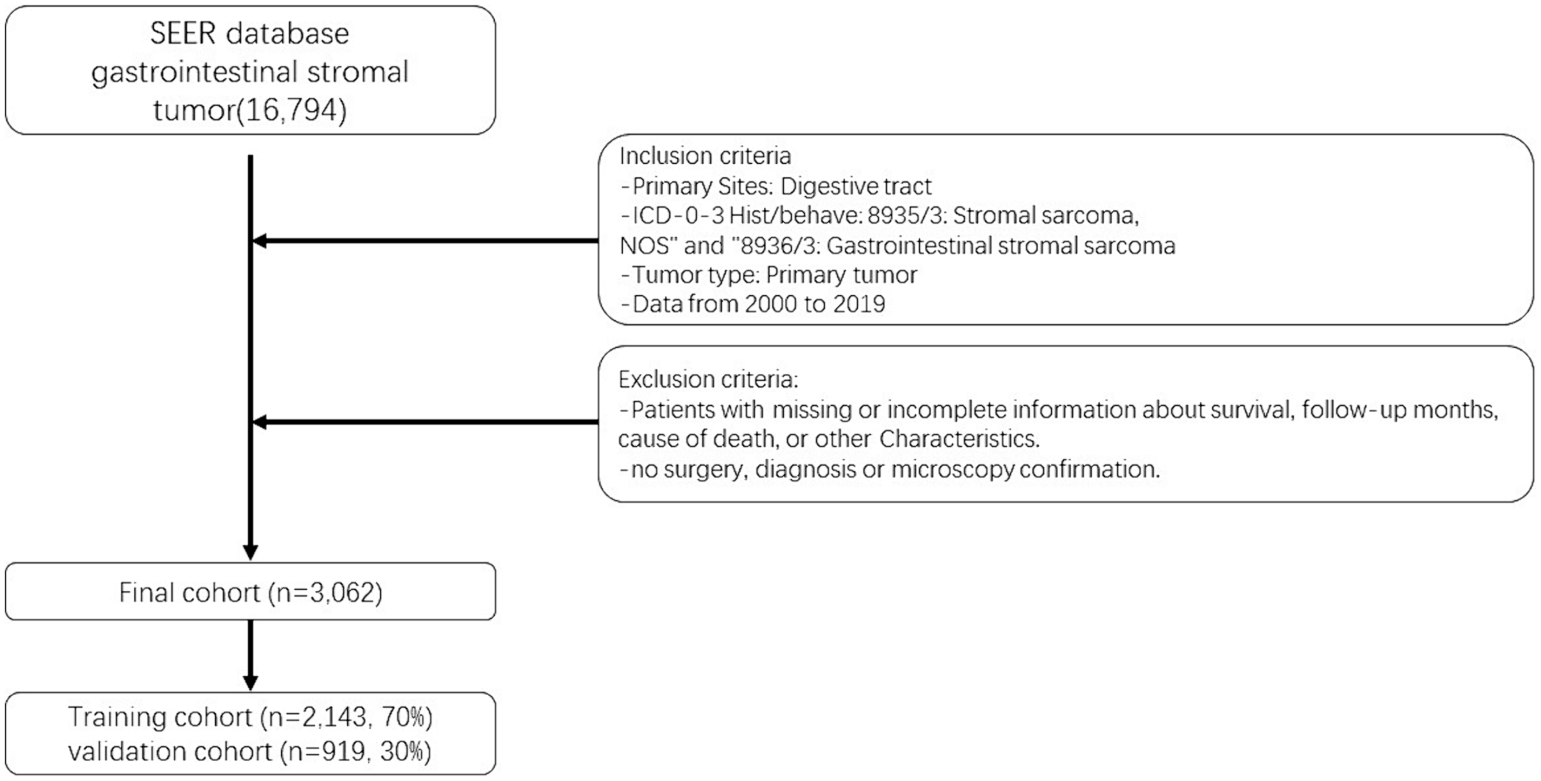
Flow chart of patient selection.

The selection of patients for analysis was based on the availability of complete baseline and survival data. The seventh edition of the AJCC staging system was adopted. Following the aforementioned methodology, an initial cohort of 16,794 GIST patients diagnosed between 2000 and 2019 was identified. After excluding patients with any missing information, a final cohort of 3,062 GIST patients was included in the study (14). To assess the model, these patients were randomly divided into a training cohort (70%) and a validation cohort (30%), with R software (version 4.2.0^1^) utilized for the analysis. Figure 1 provides a visual representation of the data screening process.

### 2.2 Nomogram and statistical analysis

The assignment of subjects to training and validation groups was followed by a log-rank test, which revealed no statistically significant difference between the groups. Baseline characteristics of each variable in the study cohort were subsequently summarized using SPSS Statistics software (version 27.0, IBM SPSS, Chicago, IL, USA). The presentation of other variable data was in the form of frequencies and percentages, while age at diagnosis was expressed as a median and interquartile range (IQR) value.

Nomograms were employed to estimate the probabilities of cancer-specific survival (CSS) at 3, 5, and 8 years for patients with GIST, and Cox regression analysis was conducted to identify factors associated with CSS in GIST (*p* = 0.05). Following the development of the nomogram, an evaluation of the model was undertaken using a set of metrics. Two metrics, namely the concordance index (C-index) and the area under the curve (AUC) of the time-dependent receiver operating characteristic (ROC), were utilized to assess the model’s discrimination capabilities. However, despite the common use of AUC and C-index, their improvements were found to be insignificant when compared to the existing model. To determine whether the new model exhibited superiority, two relatively recent metrics, the Net Reclassification Improvement (NRI) and the Integrated Discriminant Improvement (IDI), were additionally employed. While IDI considers multiple thresholds for assessing overall model performance, NRI primarily evaluates the prediction capacity of the old and new models at a specific threshold level (15, 16). These two markers are better understood and more applicable in clinical settings.

Furthermore, a calibration plot was generated to visually depict the variation between predicted and actual values. The level of model calibration indicates the consistency between predicted and observed outcomes. Improved model consistency is evident when the calibration curve aligns closely with the 45-degree reference line. Lastly, decision curve analysis (DCA) curves were employed to evaluate the clinical validity of the model. The abscissa and ordinate of the DCA curve represent the model’s threshold probability and net benefit, respectively. A higher net benefit indicates an increased utility of the model (17).

All statistical analyses were conducted using the R software package and SPSS Statistics. The fundamental characteristics of the cohort were characterized using SPSS Statistics. Subsequently, R software was utilized to randomly assign data to training and validation groups, and the log-rank test was performed. Various R packages, including survival, rms, foreign, survival ROC, nricens, and DCA packages, were employed for Cox regression analysis, proportional hazards construction testing, nomogram development, and assessment. Statistical significance was defined as two-sided probability values with *p* < 0.05.

## 3 Results

### 3.1 General characteristics

After randomizing 3,062 patients into 2 cohorts, applying the log-rank test yielded a probability value (*p* = 0.5) that indicated no significant difference between these cohorts. The fundamental demographic and clinical characteristics of the two cohorts were then described using SPSS, as shown in Table 1. In the training cohort, the median age at diagnosis was 65 years (IQR 54–77 years), while in the validation cohort, it was 64 years (IQR 55–73 years). The gender distribution and surgery were fairly even. The majority of patients in the training and validation cohorts were white (68.36 and 69.31%, respectively) and married (60.9 and 58.65%). AJCC stage I was seen in the majority of cases. The majority of patients were not treated with radiation or chemotherapy. Most patients earn about $60,000 to $74,999 a year.

**Table 1:** Demographic and clinical characteristics of the 2 cohorts of patients.

Variable	Training cohort (%)	Validation cohort (%)
*N*	2,143	919
Age of diagnosis	65 (54–74)	64 (55–73)
**Sex**		
Male	1,103 (51.47)	473 (51.47)
Female	1,040 (48.53)	446 (48.53)
**Race**		
White	1,465 (68.36)	637 (69.31)
Black	370 (17.27)	152 (16.54)
Other	308 (14.37)	130 (14.15)
**Marital status**		
Single	369 (17.22)	170 (18.5)
Married	1,305 (60.9)	539 (58.65)
DSW	469 (21.89)	210 (22.85)
**AJCC stage**		
I	1,025 (47.83)	458 (49.84)
II	373 (17.41)	145 (15.78)
III	354 (16.52)	157 (17.08)
IV	391 (18.25)	159 (17.3)
**Summary of Stage**		
Localized	1,517 (70.79)	664 (72.25)
Regional	255 (11.9)	101 (10.99)
Distant	371 (17.31)	154 (16.76)
**Radiation**		
Yes	8 (0.37)	10 (1.09)
No/unknow	2,135 (99.63)	909 (98.91)
**Chemotherapy**		
Yes	960 (44.8)	383 (41.68)
No/unknow	1,183 (55.2)	536 (58.32)
**Surgery**		
Yes	1917 (89.45)	831 (90.42)
No/unknow	226 (10.55)	88 (9.58)
**Income**		
< $35,000, $35,000–$44,999	212 (9.89)	66 (7.18)
$45,000–$59,999	472 (22.03)	221 (24.05)
$60,000–$74,999	775 (36.16)	338 (36.78)
$75,000+	684 (31.92)	294 (31.99)

### 3.2 Constructing a nomogram using the training cohort

Following a multivariate Cox stepwise regression analysis (*p* < 0.05), nine variables were identified as significant factors. These variables, along with their hazard ratios (HR) and *p*-values, are presented in Table 2. Age at diagnosis demonstrated a significant association with cancer prognosis (HR 1.051, *p* < 0.0001), while sex exhibited a protective effect (HR 0.667, *p* < 0.0001). The variable of race revealed a higher hazard ratio for black individuals compared to white individuals (HR 1.412, *p* < 0.0001). Marital status also played a role, with married individuals having a lower hazard ratio compared to those who were single (HR 0.688, *p* < 0.0001). The AJCC stage variable demonstrated notable associations with disease prognosis. Specifically, AJCC stage II had a higher hazard ratio compared to AJCC stage I (HR 1.249, *p* = 0.0608), AJCC stage III exhibited a significantly increased hazard ratio (HR 2.408, *p* < 0.0001), and AJCC stage IV showed the highest hazard ratio (HR 3.247, *p* < 0.0001) when compared to AJCC stage I.

**Table 2:** Selected variables by multivariate Cox stepwise regression analysis.

	Multivariate analysis		
Variable	HR	95% CI	*p*-value
Age of diagnosis	1.051	1.045–1.057	<0.0001
**Sex**			
Male	reference		
Female	0.667	0.579–0.768	<0.0001
**Race**			
White	reference		
Black	1.412	1.19–1.676	<0.0001
Other	0.941	0.757–1.169	0.5805
**Marital status**			
Single	reference		
Married	0.688	0.571–0.83	<0.0001
DSW	0.987	0.801–1.215	0.8997
**AJCC stage**			
I	reference		
II	1.249	0.99–1.576	0.0608
III	2.408	1.952–2.971	<0.0001
IV	3.247	2.259–4.668	<0.0001
**Summary of Stage**			
Localized	reference		
Regional	1.208	0.957–1.525	0.1126
Distant	1.375	0.979–1.931	0.0658
**Radiation**			
Yes	reference		
No/unknow	0.728	0.398–1.331	0.0302
**Chemotherapy**			
Yes	reference		
No/unknow	1.484	1.27–1.734	<0.0001
**Surgery**			
Yes	reference		
No/unknow	1.979	1.648–2.376	<0.0001
**Income**			
< $35,000, $35,000–$44,999	reference		
$45,000–$59,999	0.999	0.787–1.268	0.9903
$60,000–$74,999	0.81	0.644–1.018	0.0711
$75,000+	0.71	0.558–0.905	0.0056

Treatment-related factors were also found to be significant predictors. Patients who did not receive or had unknown radiation therapy had a lower hazard ratio compared to those who underwent radiation therapy (HR 0.728, *p* < 0.0001). Similarly, patients without or with unknown chemotherapy had a higher hazard ratio than those who received chemotherapy (HR 1.484, *p* < 0.0001). Moreover, individuals who did not undergo or had unknown surgical intervention exhibited an increased hazard ratio compared to those who underwent surgery (HR 1.979, *p* < 0.0001). Income level was identified as a significant variable, with an income of $75,000 or more associated with a lower hazard ratio when compared to an income less than $35,000 or between $35,000 and $44,999 (HR 0.71, *p* < 0.0001). These findings highlight the impact of age, sex, race, marital status, AJCC stage, treatment modalities, and income level on the prognosis of patients with gastrointestinal stromal tumor (GIST).

**Figure 2:**
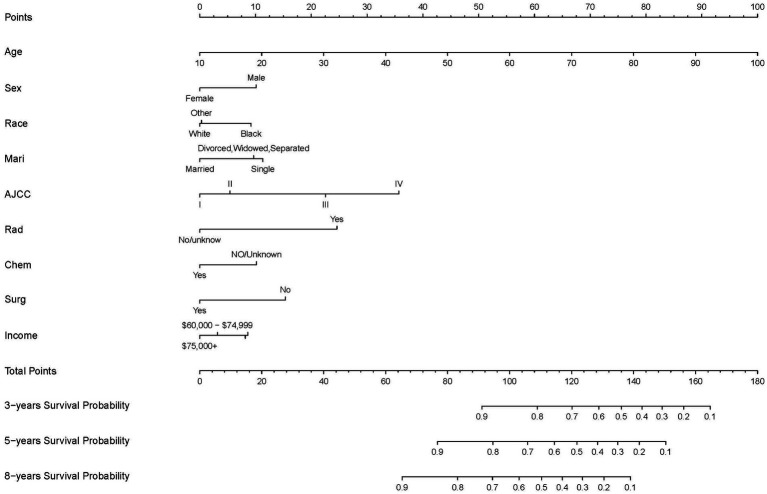
Nomogram predicting 3-, 5-, and 8-years CSS probability. Mari, marital status; Surg, surgery status; Rad, radiotherapy status; Chem, chemotherapy status.

The final nomogram, depicted in Figure 2, provides a comprehensive visualization of the multiple regression model for predicting cancer-specific survival (CSS) probabilities based on the relevant factors identified earlier. Among these factors, AJCC stage exerts the most significant influence on the survival rate, followed by surgery, race, chemotherapy, age at diagnosis, marital status, income, and sex. This figure shows scores at the top for different patient signs. Each patient’s scores are added together to get a total score. This total score matches up with the chances of dying in 3, 5, or 8 years, shown at the bottom of the nomogram. This helps doctors and patients see how likely it is that someone might die within these times. The nomogram serves as a practical tool for clinicians to estimate an individual patient’s prognosis based on the identified risk factors.

### 3.3 Evaluating the nomogram using the validation cohort

The performance of the nomogram model was assessed using the concordance index (C-index) and the area under the receiver operating characteristic (ROC) curve (AUC). In the training cohort, the C-index for the nomogram model was determined to be 0.764, indicating a good discriminatory ability to predict cancer-specific survival. Similarly, the validation cohort yielded a C-index of 0.76, further confirming the reliability of the model’s predictive capacity.

**Figure 3:**
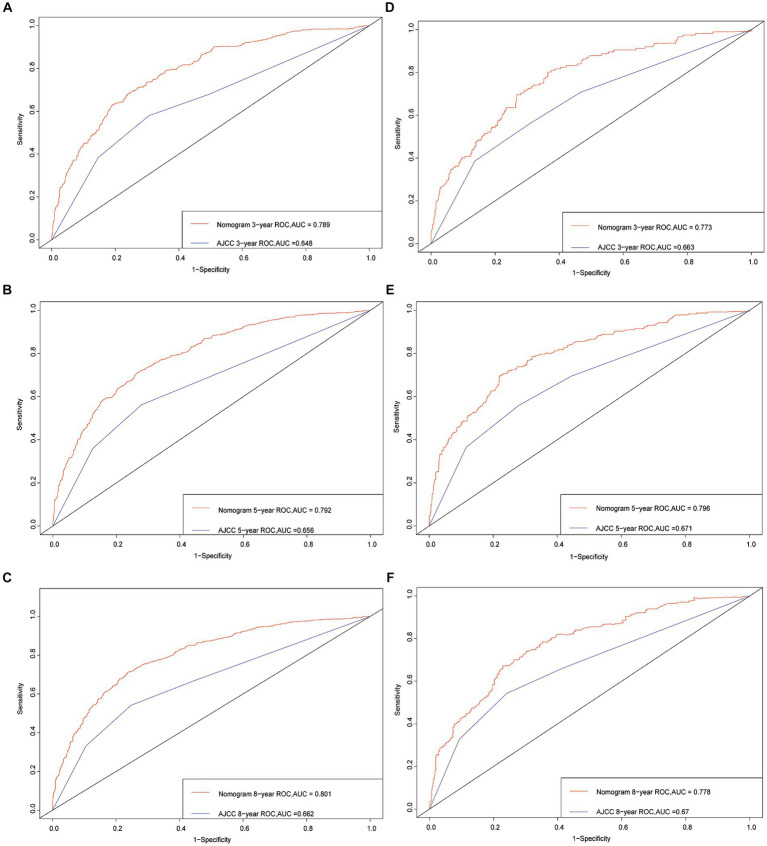
Receiver operating characteristic curves. The area under the ROC curve (AUC) for 3-, 5-, and 8-year CSS probability of the training cohort **(A–C)** and validation cohort **(D–F)**.

To evaluate the model’s discriminative power at different time points, ROC curves were constructed for 3, 5, and 8 years. The AUC values were then calculated as performance metrics. In the training cohort, the AUC values for years 3, 5, and 8 were found to be 0.789, 0.792, and 0.801, respectively. These values indicate a favorable ability of the model to differentiate between patients with different CSS probabilities at each time point. Similarly, in the validation cohort, the AUC values were 0.773, 0.796, and 0.778 for years 3, 5, and 8, respectively, further validating the model’s predictive accuracy (Figure 3).

To further assess the nomogram’s performance, additional metrics such as the Net Reclassification Improvement (NRI) and Integrated Discrimination Improvement (IDI) were employed. In the training cohort, the NRI values for the 3-, 5-, and 8-year CSS probabilities were 0.584 (95% CI 0.462–0.689), 0.641 (95% CI 0.543–0.729), and 0.663 (95% CI 0.598–0.777), respectively. Similarly, in the validation cohort, the NRI values were 0.569 (95% CI 0.347–0.721), 0.672 (95% CI 0.494–0.883), and 0.681 (95% CI 0.507–0.87). These NRI values indicate that the nomogram provides improved reclassification of patients into appropriate risk categories compared to the existing model.

The IDI values, which also assess the improvement in prediction performance, were found to be statistically significant in both the training and validation cohorts. In the training cohort, the IDI values for the 3-, 5-, and 8-year CSS probabilities were 0.095, 0.126, and 0.145, respectively (*p* < 0.001). Similarly, in the validation cohort, the IDI values were 0.091, 0.130, and 0.150 (*p* < 0.001). These results further demonstrate the enhanced predictive ability of the nomogram compared to the existing model.

**Figure 4:**
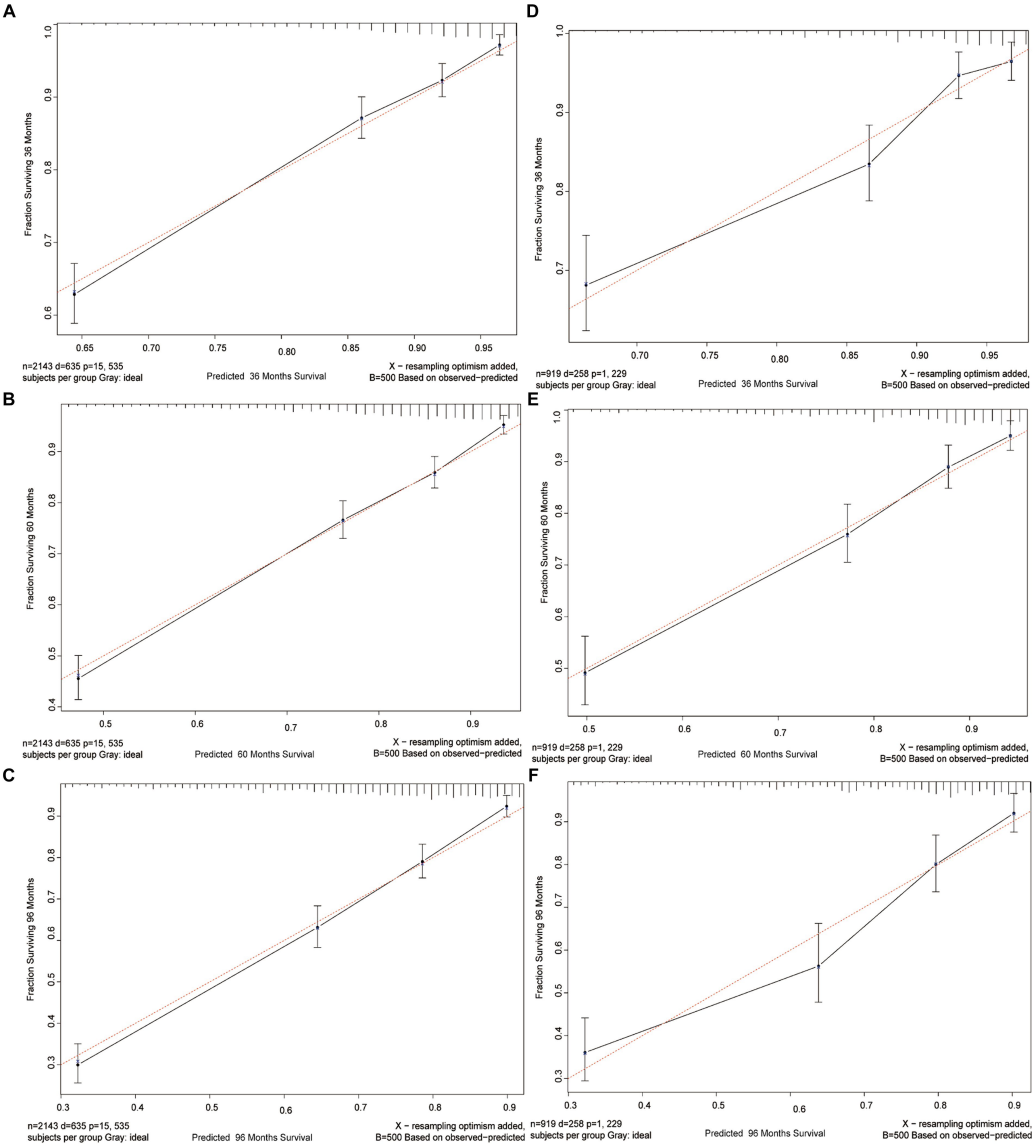
Calibration curves. Calibration curves for 3-, 5-, and 8-years CSS probability depict the calibration of each model in terms of the agreement between the predicted probabilities and observed outcomes of the training cohort **(A–C)** and validation cohort **(D–F)**.

To evaluate the calibration of the nomogram, calibration plots were generated. A calibration plot shows how closely the predictions from the nomogram match actual patient results, which is key for its use in clinical settings. The calibration plots for the 3-, 5-, and 8-year CSS probabilities in Figure 4 show a close alignment between the predicted probabilities and the ideal 45-degree reference line. This indicates a high level of calibration, suggesting that the nomogram accurately estimates the survival probabilities for GIST patients.

Overall, the NRI, IDI, and calibration plot analyses provide evidence for the nomogram’s discriminative ability, improved reclassification of patients, and accurate calibration, respectively, further supporting its reliability and clinical utility.

**Figure 5:**
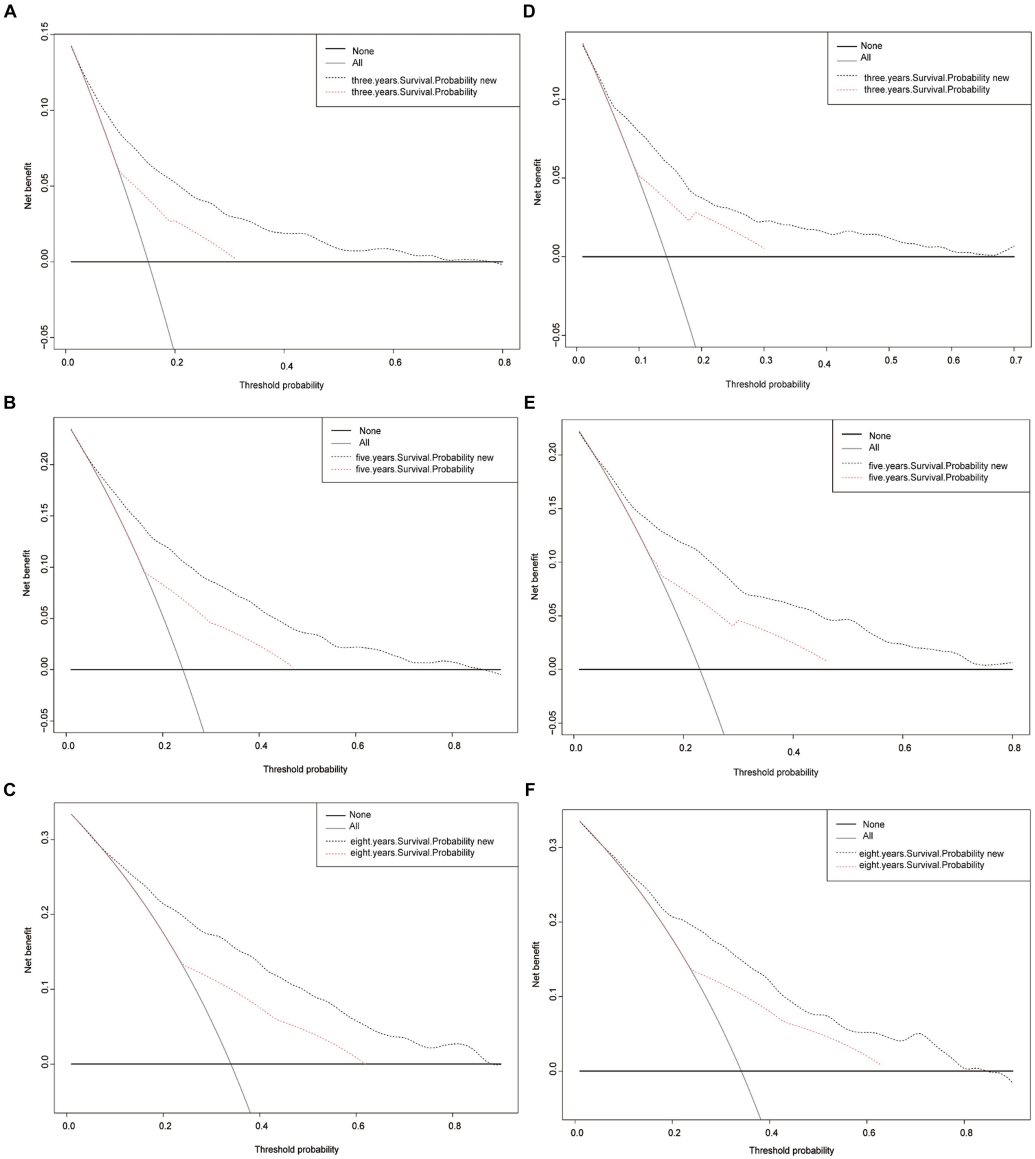
Decision curve analysis curves. Decision curve analysis of the training cohort **(A–C)** and validation cohort **(D–F)** for 3-, 5-, and 8-years CSS probability.

Finally, to assess the clinical validity of the nomogram, Decision Curve Analysis (DCA) curve was constructed. This analysis shows the range of probabilities at which the nomogram provides a net benefit, supporting its use in making clinical decisions. Figure 5 displays the survival probability curves for the new nomogram model compared to the AJCC model. Notably, the survival probability curves for the new model consistently surpass those of the AJCC model across the 3-, 5-, and 8-year time frames. This indicates that utilizing the new nomogram to predict CSS probabilities provides greater overall benefits compared to relying solely on the AJCC staging system.

## 4 Discussion

The development of a specialized nomogram for patients diagnosed with gastrointestinal stromal tumors (GIST) signifies a noteworthy stride in the realm of personalized medicine. GIST, a rare form of malignancy primarily affecting the connective tissue cells in the gastrointestinal tract, has garnered considerable attention due to its potential for aggressive behavior and the varying clinical outcomes it presents (18). However, our comprehension of GIST and its prognostic implications remains limited, underscoring the imperative for comprehensive predictive models (19).

Nomograms have become a ubiquitous tool in cancer prediction models, providing a straightforward and accurate means of estimating survival probabilities based on a range of clinical and demographic factors (9). Notwithstanding, the absence of a tailored nomogram specifically designed for GIST patients had been conspicuous prior to the inception of this study. Recognizing this void, the researchers sought to leverage the extensive data within the Surveillance, Epidemiology, and End Results (SEER) database, specifically pertaining to GIST cases, with the intention of bridging this gap and equipping clinicians with an invaluable tool for prognostication and guiding treatment decisions (20).

Backward stepwise selection in Cox regression models effectively addresses potential overfitting by iteratively removing the least significant predictors, thus simplifying the model and enhancing its generalizability. This method begins with a full model, including all potential predictors, and eliminates those with the highest *p*-values, typically based on the Wald test, indicating their low statistical significance (often using a threshold such as *p* > 0.05 or *p* > 0.10). The process continues until removing further variables would significantly worsen the model fit, assessed using criteria like the Akaike Information Criterion (AIC) or the Bayesian Information Criterion (BIC). These criteria help balance the model’s complexity against its goodness of fit, ensuring the final model is robust, not overly complex, and retains all necessary predictors for accurate predictions. This systematic reduction of predictors minimizes overfitting, making the model more applicable to new data.

The outcomes of this investigation demonstrated the successful construction and validation of a nomogram specifically tailored for predicting prognosis in GIST patients. This nomogram ingeniously incorporated nine indispensable prognostic factors, encompassing age, sex, race, marital status, American Joint Committee on Cancer (AJCC) stage, surgical status, chemotherapy status, radiation status, and income status (21, 22). These factors were meticulously identified through a backward stepwise Cox regression model, thereby illuminating their profound significance in delineating patient outcomes. The nomogram exhibited a commendable ability to discriminate, substantiated by high values of the C-index and the area under the receiver operating characteristic curve (AUC) (23, 24). The calibration curves further validated the nomogram’s reliability, while the net reclassification improvement (NRI) and integrated discrimination improvement (IDI) values unequivocally showcased its superiority over the existing AJCC model. Furthermore, the decision curve analysis (DCA) curves provided additional validation of the nomogram’s clinical utility (25). In the training cohort, the Net Reclassification Improvement (NRI) revealed noteworthy advancements in accurately classifying Conditional Survival Probability (CSS) at 3, 5, and 8 years, with increments of 58.4, 64.1, and 66.3%, respectively. Similarly, the validation cohort exhibited substantial increases of 56.9, 67.2, and 68.1% (*p* < 0.001). Another crucial metric, the Integrated Discrimination Improvement (IDI), complements the NRI by considering diverse threshold values and reflecting the overall enhancement of the model. The IDI values further substantiate that the novel model surpasses the AJCC model in predictive efficacy for CSS probabilities at 3, 5, and 8 years. Specifically, the new model demonstrates improvements of 9.5, 12.6, and 14.5% in the training cohort, and 9.1, 13, and 15% in the validation cohort (*p* < 0.001).

The development of this nomogram represents a substantial contribution to the realm of personalized medicine in the context of GIST. By assimilating essential patient characteristics and treatment approaches, the nomogram surpasses conventional methods, endowing clinicians with a comprehensive and bespoke approach to prognostication and treatment decision-making. The nomogram’s notable performance and practicality augur its potential utility in diverse clinical settings.

Nevertheless, it is incumbent upon us to acknowledge the limitations inherent in this study. The nomogram’s development and validation were based on data procured exclusively from the SEER database, which may not fully encapsulate the diversity and nuances of GIST patients encountered in real-world clinical practice. Hence, external validation is paramount to evaluate the generalizability and robustness of the nomogram. The SEER database, while a crucial resource for cancer research, has several limitations including geographic and demographic representation, as it covers only about 34.6% of the U.S. population and may not adequately represent all racial or ethnic groups. It also lacks detailed variables on lifestyle, genetics, and environmental factors, as well as comprehensive treatment data, limiting the scope of research on cancer etiology and treatment outcomes. Furthermore, it is worth noting that this study predominantly focused on cancer-specific survival (CSS) as the primary outcome variable, without considering other pivotal endpoints such as overall survival or recurrence-free survival.

## 5 Conclusion

In summary, drawing upon a substantial retrospective population, we have successfully constructed the pioneering nomogram for estimating 3-, 5-, and 8-year cancer-specific survival (CSS) probabilities in patients diagnosed with gastrointestinal stromal tumors (GIST). This innovative nomogram integrates a comprehensive array of demographic and clinicopathological characteristics. Rigorous validation and assessment protocols have underscored the utility and user-friendliness of this model, empowering physicians with a valuable resource to inform their clinical decision-making for individual patients. Notably, the nomogram has demonstrated its capacity to provide meaningful and advantageous recommendations. Moving forward, our aspirations encompass the development of more intricate nomograms, drawing from a broader range of data sources, with the aim of further enriching the predictive capabilities of these models.

## Data availability statement

The datasets presented in this study can be found in online repositories. The names of the repository/repositories and accession number(s) can be found at: https://seer.cancer.gov.

## Author contributions

XL: Conceptualization, Methodology, Writing – review & editing, Data curation, Writing – original draft. LY: Data curation, Methodology, Writing – review & editing. QL: Data curation, Methodology, Writing – review & editing. WH: Data curation, Writing – review & editing. XC: Data curation, Writing – review & editing. WG: Conceptualization, Methodology, Supervision, Writing – review & editing.

## Footnotes

^1^http://www.r-project.org
